# Submersion and hypoxia inhibit alveolar epithelial Na^+^ transport through ERK/NF-κB signaling pathway

**DOI:** 10.1186/s12931-023-02428-z

**Published:** 2023-04-24

**Authors:** Wei Zhou, Yapeng Hou, Tong Yu, Tingyu Wang, Yan Ding, Hongguang Nie

**Affiliations:** Department of Stem Cells and Regenerative Medicine, College of Basic Medical Science, China Medical University, Shenyang, China

**Keywords:** Hypoxia, Alveolar epithelial cell, Epithelial sodium channel, Extracellular signal-regulated kinase, Nuclear factor κB

## Abstract

**Background:**

Hypoxia is associated with many respiratory diseases, partly due to the accumulation of edema fluid and mucus on the surface of alveolar epithelial cell (AEC), which forms oxygen delivery barriers and is responsible for the disruption of ion transport. Epithelial sodium channel (ENaC) on the apical side of AEC plays a crucial role to maintain the electrochemical gradient of Na^+^ and water reabsorption, thus becomes the key point for edema fluid removal under hypoxia. Here we sought to explore the effects of hypoxia on ENaC expression and the further mechanism related, which may provide a possible treatment strategy in edema related pulmonary diseases.

**Methods:**

Excess volume of culture medium was added on the surface of AEC to simulate the hypoxic environment of alveoli in the state of pulmonary edema, supported by the evidence of increased hypoxia-inducible factor-1 expression. The protein/mRNA expressions of ENaC were detected, and extracellular signal-regulated kinase (ERK)/nuclear factor κB (NF-κB) inhibitor was applied to explore the detailed mechanism about the effects of hypoxia on epithelial ion transport in AEC. Meanwhile, mice were placed in chambers with normoxic or hypoxic (8%) condition for 24 h, respectively. The effects of hypoxia and NF-κB were assessed through alveolar fluid clearance and ENaC function by Ussing chamber assay.

**Results:**

Hypoxia (submersion culture mode) induced the reduction of protein/mRNA expression of ENaC, whereas increased the activation of ERK/NF-κB signaling pathway in parallel experiments using human A549 and mouse alveolar type 2 cells, respectively. Moreover, the inhibition of ERK (PD98059, 10 µM) alleviated the phosphorylation of IκB and p65, implying NF-κB as a downstream pathway involved with ERK regulation. Intriguingly, the expression of α-ENaC could be reversed by either ERK or NF-κB inhibitor (QNZ, 100 nM) under hypoxia. The alleviation of pulmonary edema was evidenced by the administration of NF-κB inhibitor, and enhancement of ENaC function was supported by recording amiloride-sensitive short-circuit currents.

**Conclusions:**

The expression of ENaC was downregulated under hypoxia induced by submersion culture, which may be mediated by ERK/NF-κB signaling pathway.

## Introduction

As the first-line between the internal and external environment, the lung regulates an optimal oxygen level in the human body, notably, the alveolar epithelial cell (AEC) is more susceptible to hypoxic condition [1]. Hypoxia is associated with many respiratory diseases including acute lung injury/acute respiratory distress syndrome (ALI/ARDS), which is characterized by the dysfunction of alveolar-capillary barrier, and the excessive edema fluid/thick mucus limits the diffusion of respiratory gases [2, 3]. The latter is partly attributable to impaired ion transport, especially epithelial sodium channel (ENaC) and Na,K-ATPase. Na^+^ is transported into AEC by ENaC at the apical surface and then pumped out by Na,K-ATPase from the basolateral surface into the interstitial and pulmonary circulation, which maintains intracellular ion homeostasis and improves alveolar fluid clearance (AFC), eventually increasing the efficiency of gas exchange [4–7]. Stabilization of Na,K-ATPase during severe hypoxia has been evidenced, which shows a HIF-dependent process [8]. Under hypoxic condition, cells attempt to adapt to environmental changes by increasing the efficiency of energy production and reducing the consumption. The activity of Na,K-ATPase, a key process of energy expenditure, is limited so as to maintain sufficient ATP levels [9, 10].

During hypoxia, extracellular signal-regulated kinase (ERK) and transcription factor nuclear factor κB (NF-κB) are activated, which are likely associated with angiogenesis, cell growth, and elaboration of proinflammatory mediators [11–13]. Moreover, hypoxia can disrupt the balance of ion transport, which in turn decreases fluid reabsorption and exacerbates pulmonary edema [14]. The activation of the ERK/AMPK/JNK axis was proved to regulate polyubiquitination of β-ENaC at the Thr615 residue and promote endocytosis of α/β-ENaC complex in AEC [15]. Furthermore, it has previously been demonstrated that phosphorylated ERK was a negative regulator for α/β/γ-ENaC mRNA expression [16]. Meanwhile, the activation of NF-κB pathway might lead to decline of α-ENaC mRNA by downregulating serine-threonine kinase expression, which was probably related to proinflammatory stimuli [17]. As important regulatory kinases of ENaC, whether ERK/NF-κB signaling pathway mediates ENaC dysregulation in the accumulation of alveolar edema fluid under hypoxia still needs further investigation.

Various physical/chemical hypoxia models have been established, one of which was to add excess volume of culture medium on the surface of respiratory epithelial cells, simulating the hypoxic environment in the state of pulmonary edema as previously described in our lab [18, 19]. AEC plays a crucial role in controlling alveolar surface fluid and epithelial ion transport, mainly by altering ENaC activity in the apical membranes of epithelial cells [4]. Breathing of newborns depends primarily on ENaC, which transports the fluid from alveolar spaces to interstitium [20, 21]. In this study, we applied the submersion culture model in human A549 and mouse fetal alveolar type 2 (AT2) cells to simulate the hypoxic environment of alveoli, which was identified by hypoxia-inducible factor 1 (HIF-1), an important index for the establishment of hypoxia [18, 22]. The aim was to explore the effects of hypoxia on ENaC expression and involved mechanism in AEC, and provide a novel strategy for treating hypoxia related pulmonary diseases.

## Materials and methods

### Cell culture and hypoxia model

A549 cells were purchased from American Type Culture Collection (ATCC, Manassas, USA) and cultured in RPMI 1640 medium (Corning, New York, USA) supplemented with 10% fetal bovine serum (Gibco, New York, USA), and 1% penicillin/streptomycin at 37 °C in humidified air containing 5% CO_2_.

AT2 cells were isolated from newborn Kunming mice within 24 h of birth. All experimental protocols involving animals were performed in accordance with the guidelines and regulations of Animal Welfare and Ethics Committee of China Medical University (No. CMU2020088). The lung tissue was separated and cut into pieces in prechilled PBS, digested with 0.25% trypsin and 0.1% type I collagenase (Sigma-Aldrich, Milwaukee, USA) at 37 °C for 40 min. Next, cells were filtered and collected, and then cultured in DMEM/F12 (Corning, New York, USA) plus 10% fetal bovine serum and 1% penicillin/streptomycin for 40 min. Unattached cells were transferred to a culture dish, repeated the above culture process for four times to remove lung fibroblasts. After that, cell collections were transferred to an IgG-coated culture dish and incubated for 1 h to remove immunocytes. Finally, the unattached cells were seeded with 2 ml culture medium in the 6-well plate, then changed every 48 h and cultured 5 d for later use.

For hypoxia model establishment on cells, excessive culture medium (8 ml) was applied to the surface of A549 and mouse AT2 cells seeded in 6-well plates, respectively. Cells were treated with 10 µM PD98059 (ERK inhibitor) (Beyotime, Shanghai, China) or 100 nM QNZ (NF-κB specific inhibitor) (MedChemExpress, Shanghai, China), submersion, or their combination and incubated for 24 h.

The exposure of hypoxia to mice (7–8 weeks old) was carried out in the chamber, in which the concentration of oxygen was 8% (Biospherix, New York, USA) for 24 h, while the normoxic Control group was processed in a normal O_2_ concentration (21%).

### Air-liquid cell culture and submersion

H441 cell (ATCC, Manassas, USA) monolayers were cultured on 24-well Transwell inserts (3413, Corning-Costar, Lowell, USA) as described previously [23]. Cells were seeded at a density about 5 × 10^6^ cells/cm^2^, then cultured in a humidified atmosphere of 5% CO_2_ at 37 °C. H441 cells were maintained in proliferation medium with RPMI 1640 containing 10% FBS, 1% P/S, and 250 nM Dexamethasone (Sigma, St. Louis, USA). Cells were allowed to attach for 24 h to reach confluence in Transwell inserts, and changed to air-liquid interface culture mode after that by removing non-adherent cells in the apical compartment. Transepithelial electrical resistance higher than 500 Ω cm^2^ had the feasibility to measure Isc levels of transepithelium in highly polarized monolayers. For hypoxia model group, 150 µl medium was applied to the apical compartment for 6 h once the cell monolayers were formed, and the air-liquid interface culture mode was used as Control. Meanwhile, 100 nM QNZ was applied to the culture medium in Hypoxia + QNZ group.

### Western blot analysis

Whole-cell lysates were prepared using RIPA containing protease and phosphatase inhibitors (Beyotime, Shanghai, China). The protein was quantified with BCA kit (Solarbio, Beijing, China), separated on a 10% SDS-polyacrylamide gel electrophoresis and transferred onto 0.45 μm PVDF membranes (Invitrogen, Carlsbad, USA). Membranes were blocked using 5% bovine serum albumin for 1 h at room temperature and incubated with primary antibodies at 4 °C overnight After washed three times by TBST for 10 min intervals, membranes were incubated with diluted secondary antibodies for 1 h at room temperature. The expression of proteins was assessed using an ECL kit (Tanon, Shanghai, China), and quantified by the image J software. The expression of β-actin did not change significantly either under hypoxia or after drug treatments, which was used as an internal parameter in our experiment accordingly.

The primary antibodies used were antibodies against α-ENaC (1:2000, Invitrogen, Carlsbad, USA), γ-ENaC (1:2000, Abcam, Cambridge, USA), β-actin (1:2000, Proteintech, Chicago, USA), HIF-1α (1:1000, Affinity Biosciences, Cincinnati, USA), ERK1/2, p-ERK1/2 (1:1000, Cell Signaling, Mass, USA), inhibitor κBα (IκBα), p-IκBα, p65, and p-p65 (1:1000, Abmart, Shanghai, China). The secondary antibodies used were goat anti-rabbit or goat anti-mouse antibodies (1:5000, ZSGB-bio, Beijing, China), respectively.

### RNA isolation and quantitative real-time PCR analysis

Total RNA was extracted by Trizol reagent (Invitrogen, Carlsbad, USA) as the manufacturer’s instructions, and the concentration was detected by NanoDrop 2000 C spectrophotometer (Thermo, Wilmington, USA). Quantitative RNA as a template was synthesized into cDNA using the PrimeScript RT reagent Kit with gDNA Eraser (TaKaRa, Kusatsu, Japan), at 37 °C for 15 min, and 85 °C for 5 s. Quantitative real-time PCR (qRT-PCR) was applied using SYBR Premix Ex Taq II (TaKaRa, Kusatsu, Japan), and the primer sequences were as follows: Mouse: α-ENaC forward (5′-AGG GCT GAG CCT AGA GCT AGA GA-3′) and reverse (5′-TTC CTC CCG GAC TGT TTG AC-3′), β-actin forward (5′-GGC TGT ATT CCC CTC CAT CG-3′) and reverse (5′-CCA GTT GGT AAC AAT GCC ATG T-3′); Human: α-ENaC forward (5′-ACT CTC TGC CGG CTA CTC AC-3′) and reverse (5′-ACC ATC GTG ACA GAA GGA GAC T-3′), β-ENaC forward (5′-ACG ACA CCC AGT ATA AGA TGA CC-3′) and reverse (5′-CCA GGT TAG AGA GCA GCC AC-3′), γ-ENaC forward (5′-GCC ACA GAT GAC CCA CTT CA-3′) and reverse (5′-TTG CGG AAC ACC ATT TGC AG-3′), and β-actin forward (5′-GT GAA GGT GAC AGC AGT CGG TT-3′) and reverse (5′-GAA GTG GGG TGG CTT TTA GGA-3′). Polymerase chain reaction for all primers was performed in the Applied Biosystems 7500 System. Relative expression of RNA was analyzed by 2^−Δ(ΔCT)^, and *Actb* (β-actin) was used as an internal reference.

### Immunofluorescence staining

After fixed with 4% paraformaldehyde for 20 min at room temperature, mouse AT2 cells were first permeabilized with 0.2% Triton X-100. Secondarily, cells were blocked with 5% bovine serum albumin in PBS for 1 h and incubated with α-ENaC primary antibody (1:200) at 4 °C overnight, then visualized by secondary antibodies for 90 min. Finally, the cell nucleus was stained by DAPI for 15 min, and observed under a fluorescence microscope.

### AFC measurement in vivo

The mice were anesthetized and the trachea was fully exposed on a 37 °C thermostatic water bath, and the mouse respirator catheter was carefully inserted into the trachea to keep the mouse breathing. Mice were placed in a left-lateral position, and 0.2 ml 5% fatty acid free BSA solution was injected into the lungs *via* the tracheal cannula completely. The respiratory rate and state of mice were observed. The alveolar fluid was aspirated after 30 min, and the protein concentration was determined with Coomassie Brilliant Blue G-250. AFC was calculated by the formula: AFC = (Vi-Vf)/Vi × 100, Vi: the initial volume of instilled solution; Vf: the volume of collected fluid, which was calculated by the formula: Vf = (Vi × Pi)/Pf, Pi/Pf: the protein contents of the initial/final alveolar fluid.

### Lung wet/dry weight ratio measurement

The left lung was isolated and weighed in an automatic electric balance (wet weight), then put in a dryer at 70 °C for 48 h and weighed again to obtain lung dry weight. The ratio of lung wet and dry weight (W/D) was calculated.

### Air-liquid cell culture and submersion

H441 cell (ATCC, Manassas, USA) monolayers were cultured on 24-well Transwell inserts (3413, Corning-Costar, Lowell, USA) as described previously [23]. Cells were seeded at a density about 5 × 10^6^ cells/cm^2^, then cultured in a humidified atmosphere of 5% CO_2_ at 37 °C. H441 cells were maintained in proliferation medium with RPMI 1640 containing 10% FBS, 1% P/S, and 250 nM Dexamethasone (Sigma, St. Louis, USA). Cells were allowed to attach for 24 h to reach confluence in Transwell inserts, and changed to air-liquid interface culture mode after that by removing non-adherent cells in the apical compartment. Transepithelial electrical resistance higher than 500 Ω cm^2^ had the feasibility to measure Isc levels of transepithelium in highly polarized monolayers. For hypoxia model group, 150 µl medium was applied to the apical compartment for 6 h once the cell monolayers were formed, and the air-liquid interface culture mode was used as Control. Meanwhile, 100 nM QNZ was applied to the culture medium in Hypoxia + QNZ group.

### Ussing chamber analysis

Either the dissected mouse tracheal epithelium or H441 cell monolayers were installed in Ussing chamber (Physiologic Instruments, San Diego, CA, USA), then bathed in solutions with (mM) 120 NaCl, 25 NaHCO_3_, 3.3 KH_2_PO_4_, 0.83 K_2_HPO_4_, 1.2 CaCl_2_, 1.2 MgCl_2_, and 10 HEPES, and 10 mannitol (apical)/10 glucose (basolateral) in each side of compartment. To determine whether hypoxia elicited a change in Na^+^ transport across the apical membrane of H441 cell monolayers, we established a 145 : 25 mM Na^+^ ionic gradient (apical to basolateral) across monolayers by replacing the 120 mM NaCl with equal-molar N-methyl-D-glucamine, an impermeant cation in the basolateral chamber. The pH and osmolality of apical/basolateral solutions were adjusted to 7.4 and 290–300 mOsm/kg, and both sides of the chambers were constantly aerated with 95% O_2_ and 5% CO_2_ at 37 °C. A transepithelial short-circuit current (Isc) was generated using electrodes and the transepithelial electrical resistance was measured by pulsing a 10 mV step every 10 s. For H441 cell monolayers, 100 µM amphotericin B was applied to the basolateral side of the Ussing chamber. Permeabilized monolayers equilibrate intracellular Na^+^ concentration to that of the bath solution in the basolateral compartment. After that, amiloride (ENaC specific inhibitor) was applied to the apical side, the decreased level of Isc was set as amiloride-sensitive Isc (ASI), which represented electrogenic Na^+^ movement by ENaC. The data was analyzed with the Acquire and Analyze program version 2.3.

### Statistical analysis

The experimental results were presented as the mean ± SE. We used the Levene and Shapiro-Wilk tests for normality and homoscedasticity of data. Student’s t-test or One-way analysis of variance (ANOVA) followed by Bonferroni’s test was applied to comparison of two groups. For the data did not pass the normality or homoscedasticity test, we used a non-parametric t-test (Mann-Whitney U-test). Statistical analysis was performed with Origin 8.0, and *P* < 0.05 was considered to be statistically significant.

## Results

### Hypoxia model was established in AEC

**Fig. 1:**
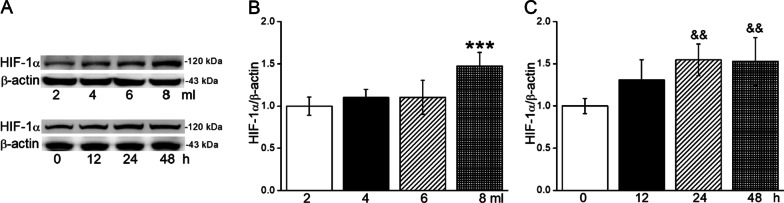
Hypoxia model was established in AEC. **A** Representative graphs of HIF-1α in different volumes (2 ml, 4 ml, 6 ml, and 8 ml) of culture medium at 24 h and HIF-1α in 8 ml at different time (12 h, 24 and 48 h) in A549 cells. **B**, **C** The statistical data obtained from Western Blot and quantified through gray analysis (HIF-1α/β-actin). ***P < 0.001, compared with 2 ml; ^&&^*P* < 0.01, compared with 0 h. One-way ANOVA followed by Bonferroni’s test was used to analyze the difference of the means for significance. n = 5–6

Different volumes at different time points were tested to find the suitable hypoxic condition in A549 cells. As the capacity of culture medium increased (2 ml, 4 ml, 6 ml, and 8 ml) at 24 h, the expression of HIF-1α in A549 cells increased volume-dependently (Fig. 1A, B). Moreover, the increased expression of HIF-1α in 8 ml culture medium showed a time-dependent manner (12 h, 24 h, and 48 h), which was obviously significant at 24 and 48 h and almost reached a plateau after 24 h, respectively, indicating the establishment of hypoxia model (Fig. 1A, C).

### Hypoxia downregulated the expression of ENaC

**Fig. 2:**
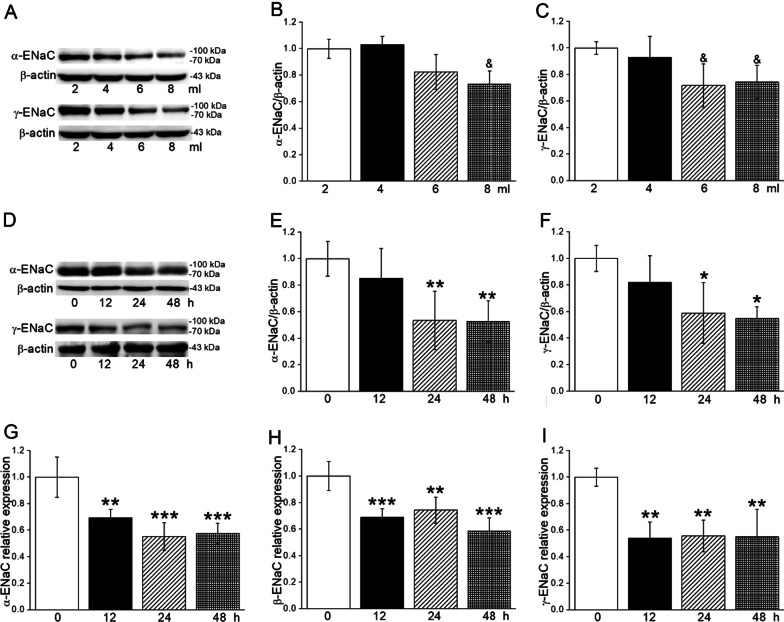
Hypoxia decreased the protein and mRNA of ENaC. **A** Representative graph of α/γ-ENaC in different volumes (2 ml, 4 ml, 6 ml, and 8 ml) of culture medium for 24 h in A549 cells. **B**, **C** The statistical data of α/γ-ENaC in different volumes (2 ml, 4 ml, 6 ml, and 8 ml) of culture medium for 24 h in A549 cells obtained from Western Blot and quantified through gray analysis (α/γ-ENaC/β-actin). **D** Representative graph of α/γ-ENaC in 8 ml culture medium at different times (12 h, 24 h, and 48 h) in A549 cells. **E**, **F** The statistical data obtained from Western Blot and quantified through gray analysis (α/γ-ENaC/β-actin). **G–I** α/β/γ-ENaC mRNA expression level of A549 cells after 12 h, 24 h, and 48 h in 8 ml culture medium (α/β/γ-ENaC/β-actin). ^&^*P* < 0.05, compared with 2 ml group. *P < 0.05, **P < 0.01, ***P < 0.001, compared with 0 h group. One-way ANOVA followed by Bonferroni’s test was used to analyze the difference of the means for significance. n = 3–6

To investigate the influence of hypoxia on alveolar epithelial ion transport, we explored the α/γ-ENaC protein expression in A549 cells, which showed a decreased tendency in both volume and time-dependent manners (Fig. 2A–F). Meanwhile, we observed a decrease of α/β/γ-ENaC mRNA expression after the administration of 8 ml culture medium (Fig. 2G–I). Accordingly, treatment of 8 ml culture medium and duration for 24 h were chosen for the following hypoxia model setup. We didn’t detect the β-ENaC expression for the lack of suitable antibody for the Western Blot assay.

### ERK/NF-κB signaling pathway was activated under hypoxia

**Fig. 3:**
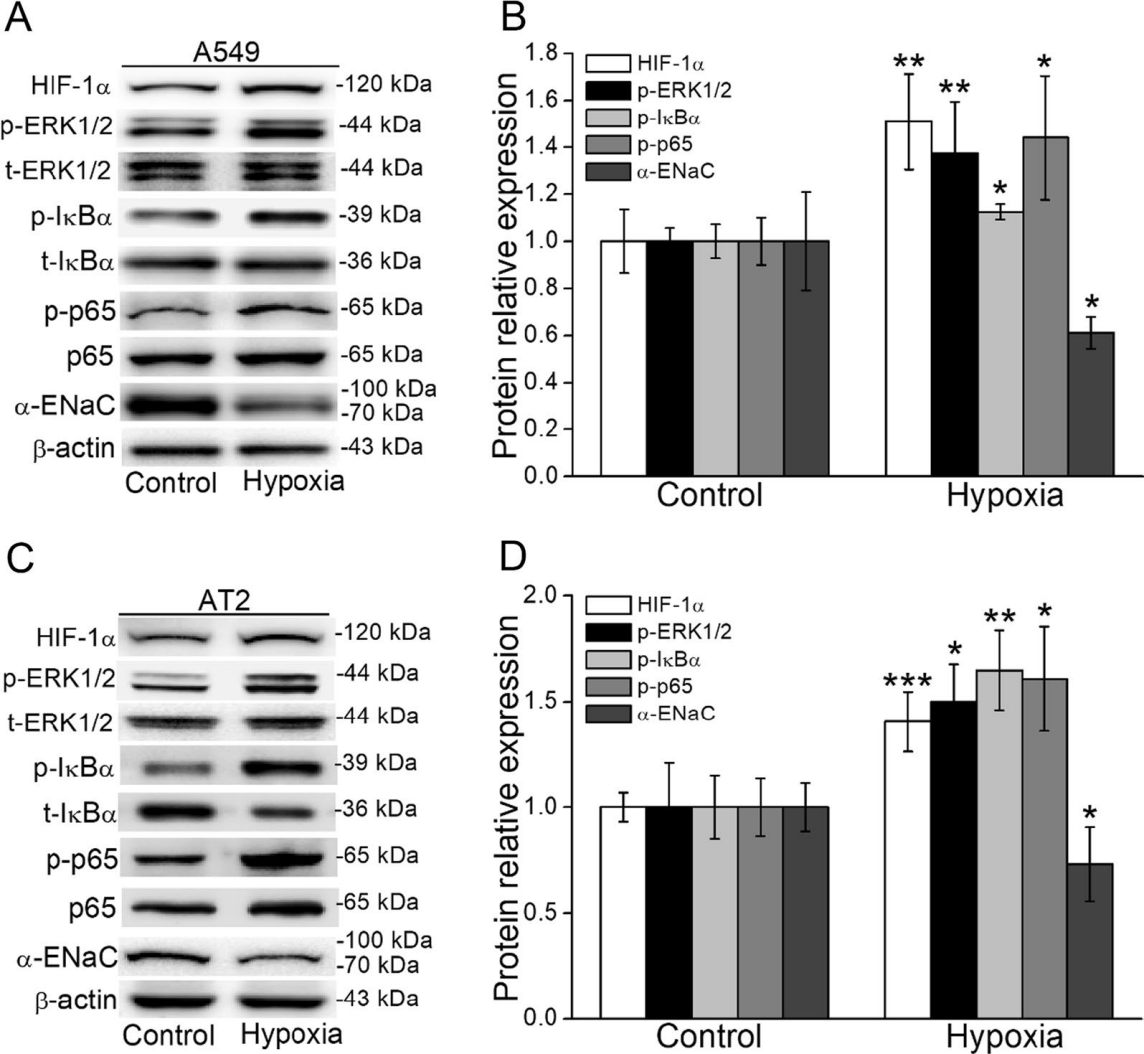
Effect of hypoxia on ENaC and associated signal pathways on A549 and mouse AT2 cells. **A**, **C** Representative graph of HIF-1α, α-ENaC, ERK1/2, and NF-κB signaling pathways under hypoxia. **B**, **D** The statistical data obtained from Western Blot and quantified through gray analysis. *P < 0.05, **P < 0.01, ***P < 0.001, compared with Control group. Mann-Whitney U test with Bonferroni correction and One-way ANOVA followed by Bonferroni’s test were used to analyze the difference of the means for significance. n = 4–6

As important regulatory kinases of ENaC, ERK and NF-κB can be activated by hypoxia [13, 24]. To observe the possible involvement of ERK/NF-κB signaling pathway in the regulation of ENaC under hypoxia, we measured both ERK phosphorylation and NF-κB in A549 and mouse AT2 cells, respectively. As shown in Fig. 3A–D, besides the expression of HIF-1α, the phosphorylated ERK, IκB, and p65 were increased, whereas α-ENaC was decreased under hypoxia.

**Fig. 4:**
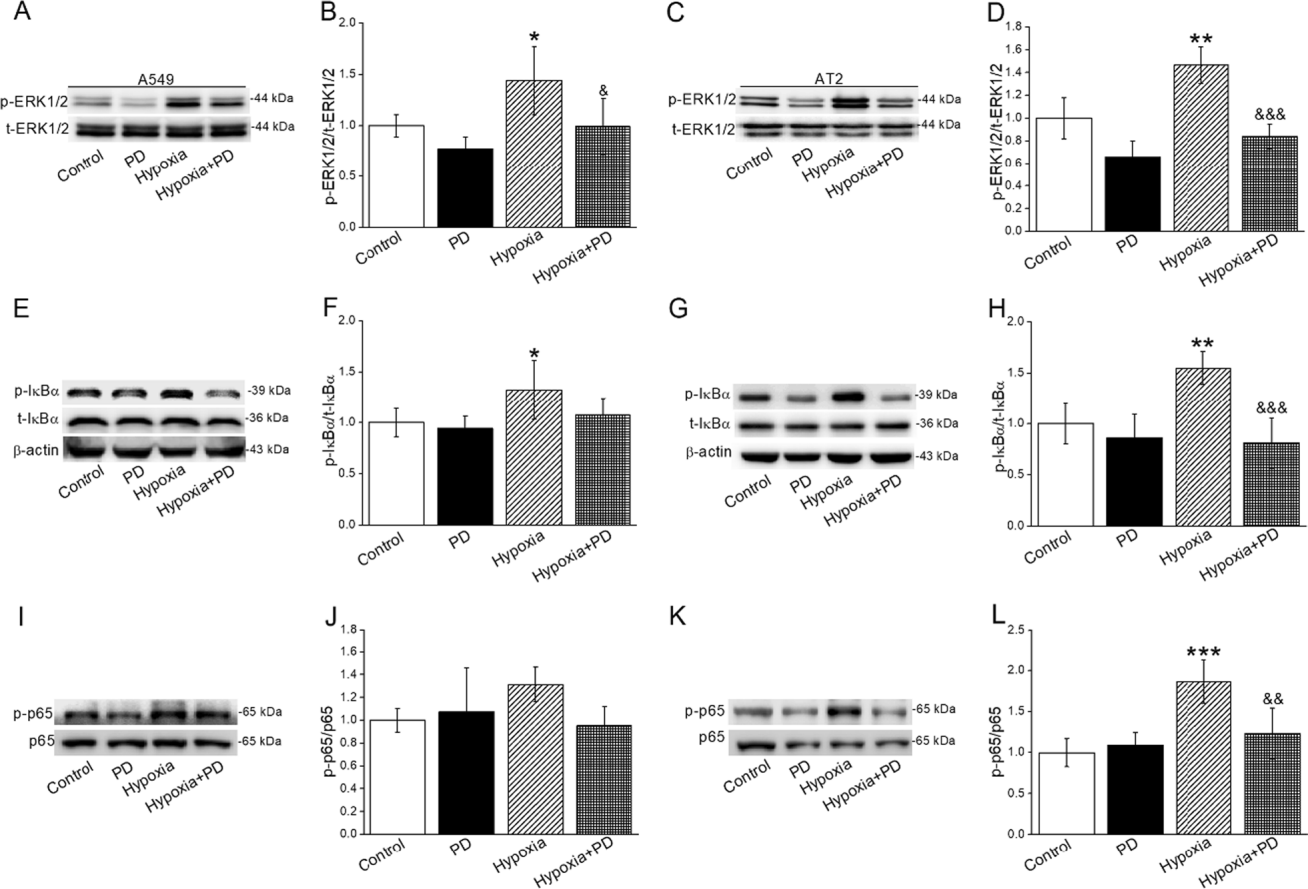
Hypoxia-induced activation of NF-κB signaling pathway was downregulated by ERK inhibitor in A549 and AT2 cells. **A**–**D** Representative and statistical data of t-ERK1/2 and p-ERK1/2 after PD98059 (ERK inhibitor, 10 µM) treatment. **E**–**H** Representative and quantitative analyses of p-IκB/t-IκB. **I–L** Representative and quantitative analyses of p-p65/p65. *P < 0.05, **P < 0.01, ***P < 0.001, compared with Control group; ^&^*P* < 0.05, ^&&^*P* < 0.01, ^&&&^*P* < 0.001, compared with Hypoxia group. One-way ANOVA followed by Bonferroni’s test was used to analyze the difference of the means for significance. n = 3–6

After the administration of ERK1/2 inhibitor (PD98059), both of the phosphorylated expressions in ERK1/2 and IκB/p65 were decreased, implying that NF-κB signaling pathway might be one of the downstream molecules of ERK1/2 under hypoxia (Fig. 4A–L).

### ERK/NF-κB was associated with downregulation of α-ENaC under hypoxia

**Fig. 5:**
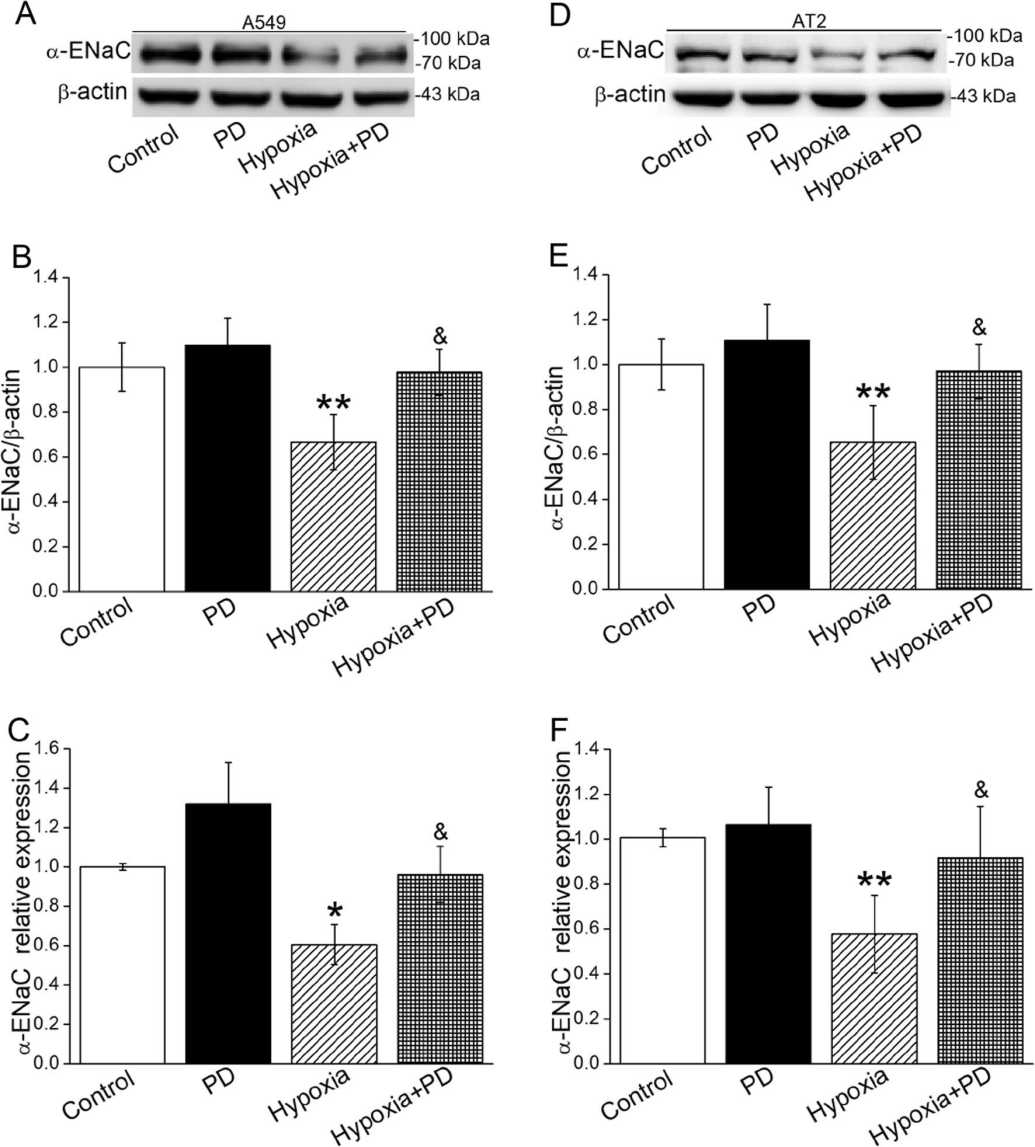
Hypoxia-induced decrease of α-ENaC was mediated by the activation of the ERK1/2 signaling pathway. **A**, **B** Representative and statistical data of Western Blot of α-ENaC expression of A549 cells cultured under hypoxia (8 ml) for 24 h after PD98059 (10 µM) treatment. **D**, **E** Representative and statistical data of Western Blot of α-ENaC expression of AT2 cells cultured in hypoxic condition (8 ml) for 24 h after PD98059 (10 µM) treatment. **C**, **F** qRT-PCR results for α-ENaC mRNA in A549 and AT2 cells, and β-actin was used as internal reference. *P < 0.05, **P < 0.01, compared with Control group; ^&^*P* < 0.05, compared with Hypoxia group. One-way ANOVA followed by Bonferroni’s test was used to analyze the difference of the means for significance. n = 3–6

**Fig. 6:**
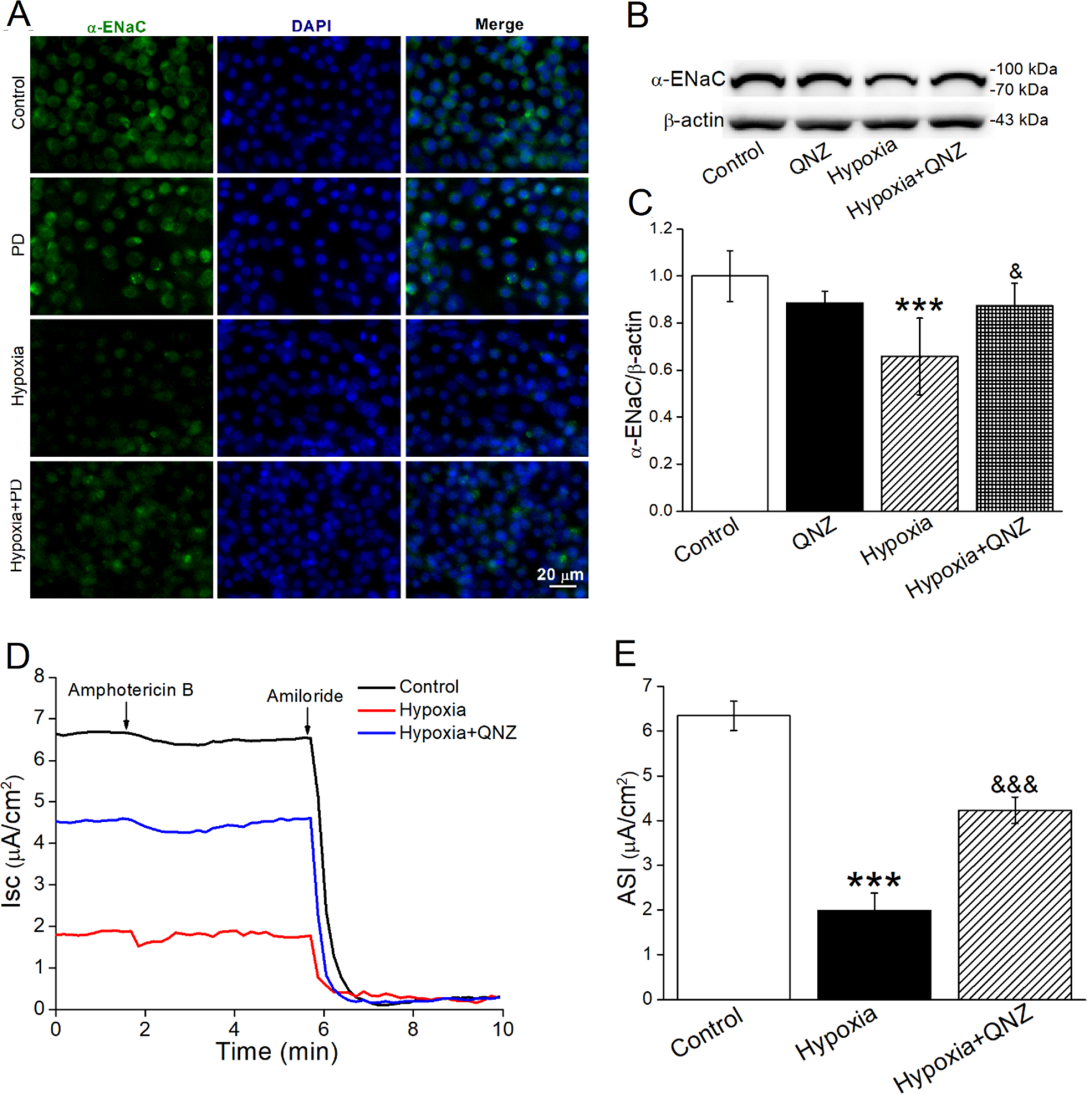
Hypoxia-induced decrease of α-ENaC was mediated by the activation of the ERK1/2 and NF-κB signaling pathways. **A** Immunofluorescence of α-ENaC (green) in AT2 cells cultured in hypoxic condition (8 ml) for 24 h after PD98059 (10 µM) treatment. The nucleus was stained with DAPI (blue). Scale bar = 20 μm. **B**, **C** Representative and statistical data of α-ENaC in AT2 cells cultured in hypoxic condition (8 ml) for 24 h after QNZ (NF-κB inhibitor, 100 nM) treatment. n = 4–6. **D** Representative Isc traces of Control, Hypoxia and Hypoxia + QNZ group in H441 cell monolayers, and amphotericin B (100 µM) was added to the basolateral compartment, followed by 100 µM amiloride applied to the apical side. **E** Statistic ASI, defined as the difference between the total current and the amiloride-resistant current, n = 4. ***P < 0.001, compared with Control group; ^&^*P* < 0.05, ^&&&^*P* < 0.001, compared with Hypoxia group. One-way ANOVA followed by Bonferroni’s test was used to analyze the difference of the means for significance

To further demonstrate whether ENaC was downregulated under hypoxia mediated by ERK and NF-κB, we detected the protein expression of α-ENaC by administration of corresponding inhibitors in both A549 and mouse AT2 cells. As shown in Figs. 5 and 6A, inhibition of ERK significantly alleviated the hypoxia downregulated α-ENaC both at protein and mRNA expression levels. The image of immunofluorescence assay for α-ENaC was consistent with the above (Fig. 6A). The γ-ENaC subunit also showed the same tendency, although in an insignificant way (data not shown).

Similarly, the protein expression of α-ENaC was increased in the case of NF-κB pathway inhibition (Fig. 6B, C). In addition, to further observe the regulation of NF-κB on ENaC function under hypoxia, we examined the Isc in permeabilized H441 cell monolayers by Ussing chamber assay with amphotericin B in the basolateral compartment. As expected, the result showed a decrease of ASI under hypoxia, which reflected ENaC function and could be reversed by NF-κB inhibitor (Fig. 6D, E), supporting that hypoxia could downregulate ENaC through ERK/NF-κB signaling pathway.

### Hypoxia downregulated AFC by NF-κB signaling pathway

**Fig. 7:**
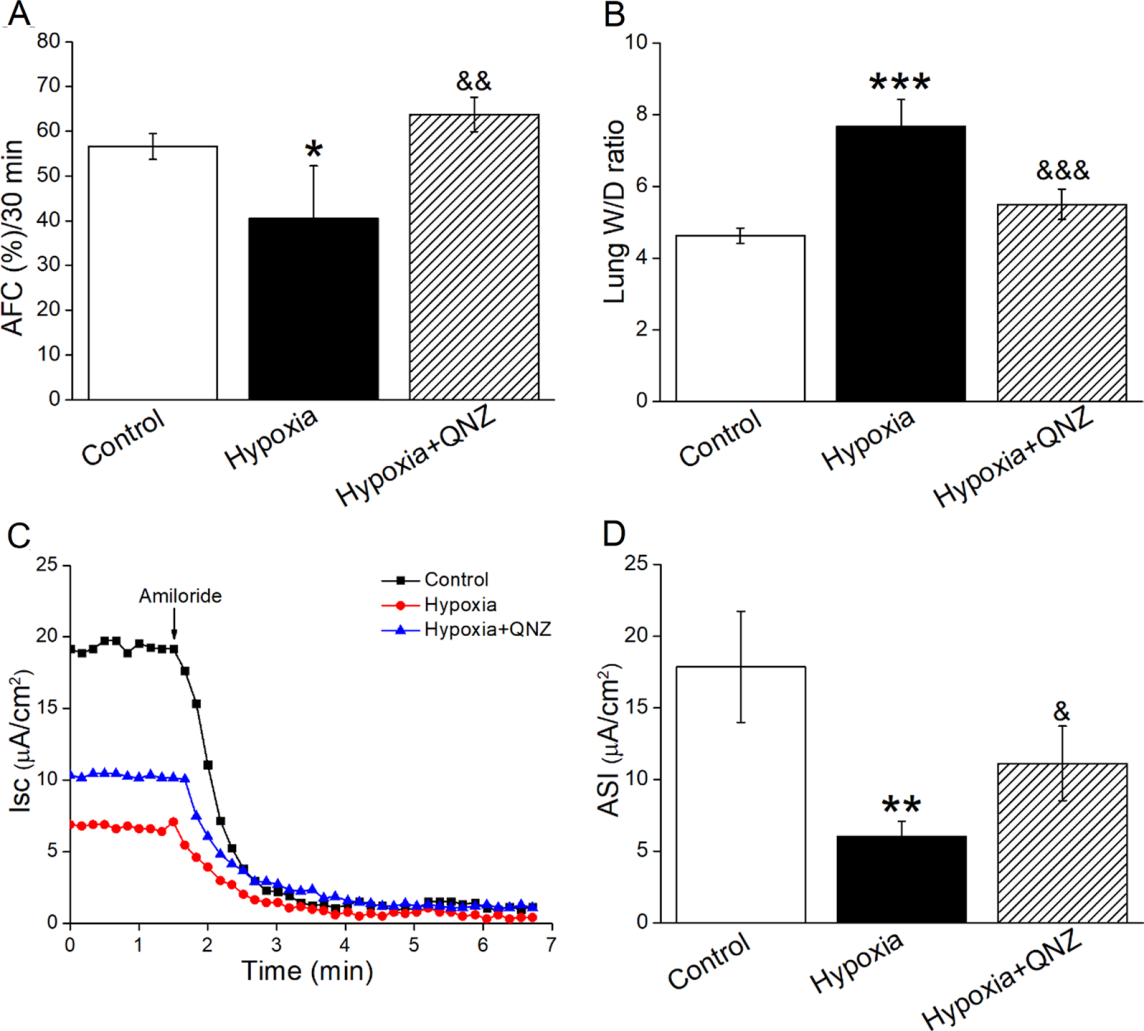
Hypoxia-induced decrease of AFC and ENaC activity were associated NF-κB pathway. **A** NF-κB inhibitor (QNZ, 1 mg/kg) was administered intraperitoneally to mice 1 h before the exposure to hypoxic condition (8% O_2_) for 24 h, and the effect of NF-κB inhibitor was assessed by AFC assay, n = 4–5. **B** The lung W/D ratio changed in QNZ-treated mice, n = 3–5. **C** Representative Isc traces of Control, Hypoxia and Hypoxia + QNZ group in mouse tracheal epithelium, and 1 mM amiloride was added to apical solution. **D** Statistic ASI, defined as the difference between the total current and the amiloride-resistant current, n = 3–4. *P < 0.05, **P < 0.01, ***P < 0.001, compared with Control group; ^&^*P* < 0.05, ^&&^*P* < 0.01, ^&&&^*P* < 0.001, compared with Hypoxia group. One-way ANOVA followed by Bonferroni’s test was used to analyze the difference of the means for significance

The above experiments have proved that ERK/NF-κB signaling pathway was involved in the downregulation of ENaC under hypoxia in vitro, next we further demonstrated the related mechanisms of hypoxia on lung function in vivo. As the result of Fig. 7A, hypoxia (8% O_2_, 24 h) downregulated AFC of mice, which was alleviated after pretreated with NF-κB inhibitor under hypoxia. Similarly, NF-κB inhibitor significantly attenuated the hypoxia increased W/D ratio, an indicator for the degree of pulmonary edema (Fig. 7B). We repeated Ussing chamber assay in the mouse tracheal epithelium and similarly, NF-κB inhibitor could strengthen ENaC function by enhancing the hypoxia reduced ASI (Fig. 7C, D).

## Discussion

**Fig. 8:**
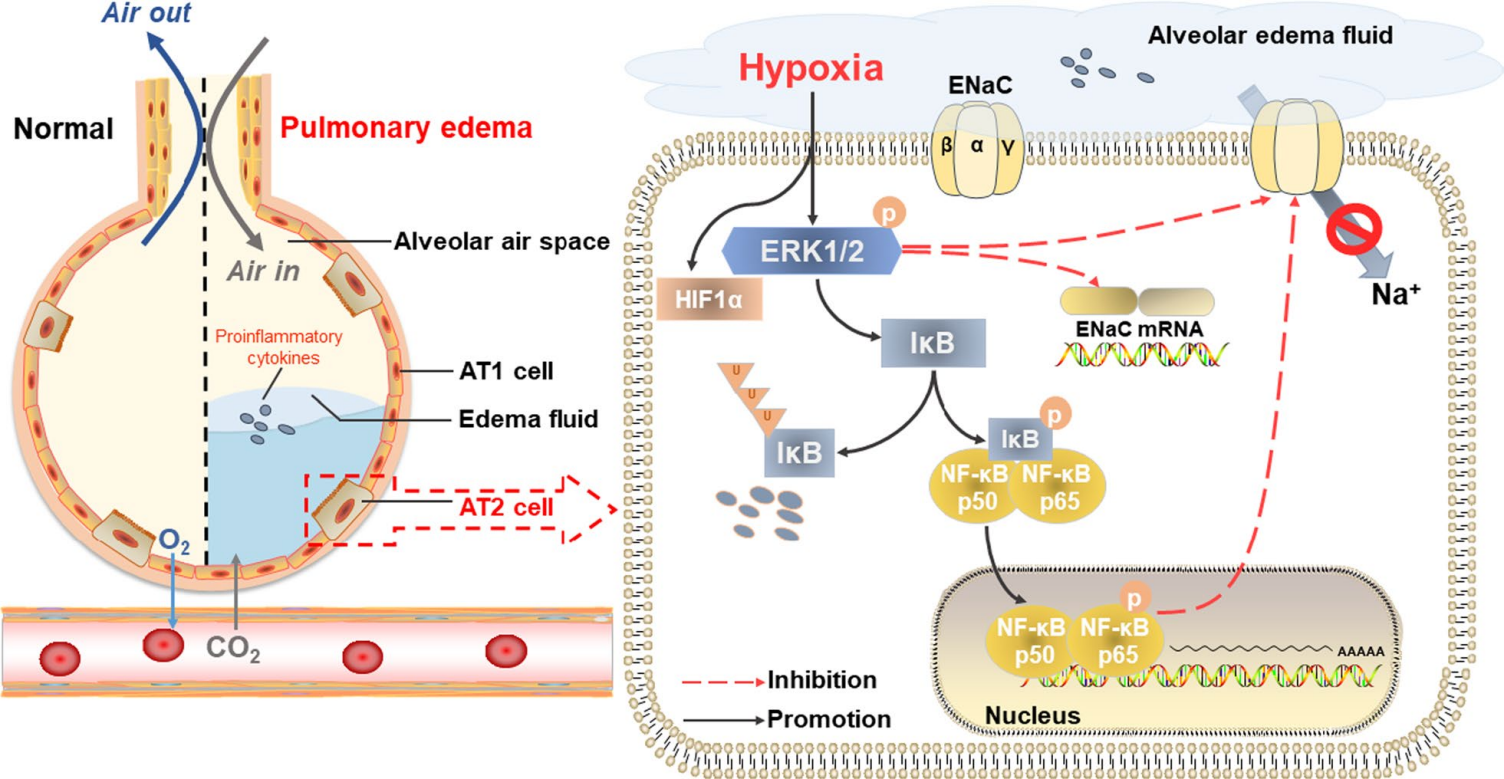
The schematic diagram depicts the potential mechanism about decreased expression of ENaC in AT2 cells during hypoxia. The accumulation of pulmonary edema fluid created a hypoxic environment, which stimulated the phosphorylation of ERK1/2, induced phosphorylation of IκB and NF-κB p65, and promoted nuclear translocation of p65. Proinflammatory cytokines may also affect ENaC activity and expression. Eventually, vectorial Na^+^ transport was suppressed, leading to edema formation. *AT1/2* alveolar type 1/2, *ERK1/2* extracellular signal-regulated kinase 1/2, *ENaC* epithelial sodium channel, *HIF-1α* hypoxia-inducible factor 1α, *IκBα* inhibitor κBα, *NF-κB* nuclear factor κB

Hypoxia is a common feature of various respiratory diseases, such as ALI/ARDS, which is mainly characterized by pulmonary edema and worsened from each other (Fig. 8) [25–27]. ENaC is a heterotrimeric ion channel mainly composed of α, β, and γ subunits in most mammalian epithelial cells, among which α subunit plays a dominant role in the absorption of alveolar edema fluid [20, 28–30]. The hypoxic environment formed by the alveolar edema fluid constitutes an important factor in the reduction of ENaC expression and activity, which in turn increases the accumulation of alveolar edema fluid [27]. Previous studies proved that hypoxia-induced inhibition of ENaC in collecting duct principal cells might be associated with HIF pathway, silence of which increased β/γ-ENaC expression [31]. Meanwhile, acute exposure of AECs to hypoxia disrupted the balance of amiloride-sensitive Na^+^ transport and reduced ENaC activity, which might be attributed to Nedd4-2-mediated ubiquitination in the apical membrane [32]. However, moderate exposure to hypoxia impaired epithelial Na^+^ transport on airway in a reversible manner, without inhibiting ENaC expression [33]. Consistent with the above, we found that submersion and hypoxia resulted in the decreased expression and function of ENaC in our experiment. However, the detailed mechanism of hypoxic environment caused by alveolar edema fluid accumulation is still unknown, here we aim to explore the relative mechanism based on both cell and animal models. As a major constitute of AEC, AT2 cells (A549 cells as a substitute for human AT2 and primary mouse AT2 cells) were selected for parallel experiments to explore the mechanism of hypoxia-induced ENaC reduction [34, 35]. Given the cavitary structure of the alveoli, which could hardly be used to record currents, we selected the tracheal epithelium of mice to better demonstrate the effect of hypoxia on ENaC activity in vivo.

We simulated the pathological state of pulmonary edema by adding excessive culture medium to the surface of AEC, which provided an alternative model to investigate the effect of hypoxia. According to Fick’s First Law, when the culture medium was increased to 8 ml, the oxygen supplement was theoretically decreased to a quarter compared with 2 ml [18, 36–39], and this submersion culture mode was confirmed as hypoxic condition by the indicator of HIF-1α [40].

In our study, hypoxia induced the ERK phosphorylation and NF-κB activation, which was consistent with previous studies [41–43]. NF-κB as a central role in the inflammation response, was involved in the regulation of HIF-1α expression. Studies have shown that ERK and NF-κB were critical transcriptional activators of HIF-1α, and that basal NF-κB activity was required for the protein accumulation of HIF-1α under hypoxia [43, 44]. Consequently, the stable expression of HIF-1α in our experiment might be partly due to the activation of ERK/NF-κB. However, some studies also showed that HIF-1α repressed NF-κB-dependent gene expression due to the competition of coactivator p300, and thus prevented overly pro-inflammatory responses [45, 46]. Intriguingly, the protein and mRNA expressions of ENaC were decreased in our hypoxia model. Inhibition of the phosphorylation of ERK could decrease the phosphorylated IκB and p65 under hypoxia, similar with NF-κB as a downstream of ERK in modulating proinflammatory cytokine genes [47, 48]. Meanwhile, the downregulated ENaC in hypoxia was associated with the increased activation of ERK and NF-κB signaling pathways.

Previous studies have proven that phosphorylated ERK1/2 is a negative regulator for ENaC either by the inhibition of α/β/γ-ENaC mRNA expression or promotion of α/β-ENaC complex endocytosis in AEC [49, 50]. In our hypoxia model, the expression of ENaC was increased after inhibiting ERK phosphorylation, which is consistent with the above and suggests that ERK could affect ENaC expression as an independent factor (Fig. 8) [15, 16, 51, 52]. Moreover, previous studies demonstrated that the elevation of α-ENaC induced by IL-1 was mediated *via* NF-κB activation, which was in part involved with the stimulation of ERK1/2 [53, 54]. Besides the effects of ERK and NF-κB, studies have implied that many other signaling pathways and kinases regulated the expression and activity of ENaC in pathologies, such as ALI/ARDS. Among them, protein kinase C and AMPK had inhibitory effects on the open probability or internalization of ENaC *via* ubiquitination [15, 52]. Additionally, HIF-1 as an important regulator of inflammation associated with IκBα and NF-κB activation, could promote alveolar macrophages to release proinflammatory cytokines (including TNF-α, IL-1β, IL-4, IL-6, and IL-17, etc.), which induced the expression of NF-κB and inducible nitric oxide synthase/nitric oxide [26, 55]. All of the above inflammatory products could ultimately downregulate the activity and surface abundance of ENaC, inhibit alveolar epithelial Na^+^ transport and edema fluid reabsorption, which were aggravated under hypoxia [56–58]. In contrast, mTORC2, PPARγ, and PI3K/AKT could enhance the expression of SGK1, eventually resulting in upregulation of ENaC and reduction of pulmonary edema [29, 49, 59].

To validate our hypothesis that ERK and NF-κB may also have a regulatory effect on ENaC under hypoxia, we applied the corresponding inhibitors and found that α-ENaC expression was increased accordingly (Fig. 8). The reason may be explained by the phenomenon that the activation of NF-κB pathway related to proinflammatory stimuli might downregulate serine-threonine kinase SGK1 expression, a stimulator of ENaC, and lead to decreased α-ENaC mRNA level consequently [13, 17, 60, 61]. Unfortunately, given that A549 cells could not be a perfect model to establish alveolar epithelial barrier due to the disability to form tight junction, we chose permeabilized H441 cell monolayer as an alternative model in studying the function of epithelial sodium transport [23, 62, 63].

Of note, there were also other possible causes of pulmonary edema under hypoxia, like dysfunction of other ion channels/transporters and tight junctions in alveolar epithelium, leading to impaired fluid absorption [3, 13]. Besides affecting the ability of fluid clearance in lung epithelium, hypoxia could also cause contraction of the vasculature, resulting in pulmonary hypertension, broken endothelium tight barrier, increased permeability, and eventually edema formation. The above might explain that in our in vivo experiment, hypoxia-inhibited AFC and enhanced lung wet/dry ratio could be partially prevented by QNZ to some different degree [64, 65]. Our study of detailed mechanism about ENaC regulation under hypoxia may solve an urgent priority in relieving pulmonary edema, which provides a possible breakthrough in treating ALI/ARDS.

## Conclusions

Hypoxia could inhibit alveolar epithelial ion transport by decreasing ENaC expression, which might be mediated by activation of ERK/NF-κB signaling pathway.

## Data Availability

The datasets used and analyzed during the current study are available from the corresponding author on reasonable request.

## Abbreviations

AEC: Alveolar epithelial cell
AFC: Alveolar fluid clearance
ALI: Acute lung injury
ARDS: Acute respiratory distress syndrome
ASI: Amiloride-sensitive short-circuit current
AT2: Alveolar type 2
ERK: Extracellular signal-regulated kinase
ENaC: Epithelial sodium channel
HIF-1: Hypoxia-inducible factor 1
Isc: Short-circuit current
IκBα: Inhibitor κBα
NF-κB: Nuclear factor κB
qRT-PCR: Quantitative real-time PCR
